# Association of Genetic Variation in the 3'UTR of LHX6, IMMP2L, and AADAC With Tourette Syndrome

**DOI:** 10.3389/fneur.2020.00803

**Published:** 2020-08-14

**Authors:** Luca Pagliaroli, Andrea Vereczkei, Shanmukha Sampath Padmanabhuni, Zsanett Tarnok, Luca Farkas, Peter Nagy, Renata Rizzo, Tomasz Wolanczyk, Urszula Szymanska, Mira Kapisyzi, Entela Basha, Anastasia Koumoula, Christos Androutsos, Vaia Tsironi, Iordanis Karagiannidis, Peristera Paschou, Csaba Barta

**Affiliations:** ^1^Institute of Medical Chemistry, Molecular Biology and Pathobiochemistry, Semmelweis University, Budapest, Hungary; ^2^Department of Molecular Biology and Genetics, Democritus University of Thrace, Alexandroupoli, Greece; ^3^Vadaskert Clinic for Child and Adolescent Psychiatry, Budapest, Hungary; ^4^Materno Infantile and Radiological Science Department, University of Catania, Catania, Italy; ^5^Department of Child Psychiatry, Medical University of Warsaw, Warsaw, Poland; ^6^University Hospital Center “Mother Theresa,” Tirana, Albania; ^7^Department of Child and Adolescent Psychiatry, Sismanoglio General Hospital of Attica, Athens, Greece

**Keywords:** 3'UTR, genetic variation, microRNA, SNP, Tourette Syndrome

## Abstract

**Background:** Tourette Syndrome (TS) is a neurodevelopmental disorder that presents with motor and vocal tics early in childhood. The aim of this study was to investigate genetic variants in the 3' untranslated region (3'UTR) of TS candidate genes with a putative link to microRNA (miRNA) mediated regulation or gene expression.

**Methods:** We used an *in silico* approach to identify 32 variants in the 3'UTR of 18 candidate genes putatively changing the binding site for miRNAs. In a sample composed of TS cases and controls (*n* = 290), as well as TS family trios (*n* = 148), we performed transmission disequilibrium test (TDT) and meta-analysis.

**Results:** We found positive association of rs3750486 in the LIM homeobox 6 (LHX6) gene (*p* = 0.021) and rs7795011 in the inner mitochondrial membrane peptidase subunit 2 (IMMP2L) gene (*p* = 0.029) with TS in our meta-analysis. The TDT showed an over-transmission of the A allele of rs1042201 in the arylacetamide deacetylase (AADAC) gene in TS patients (*p* = 0.029).

**Conclusion:** This preliminary study provides further support for the involvement of LHX6, IMMP2L, and AADAC genes, as well as epigenetic mechanisms, such as altered miRNA mediated gene expression regulation in the etiology of TS.

## Introduction

Tourette Syndrome (TS) is a neurodevelopmental disorder characterized by the presence of vocal and motor tics. Most cases present with comorbid conditions, such as obsessive-compulsive disorder (OCD) and attention deficit hyperactivity disorder (ADHD), but also with anxiety, behavioral disorders, autism spectrum disorders, and learning disabilities (1).

Tourette Syndrome has a complex etiology, both genetic and environmental factors are believed to contribute to its development. Several external factors, such as perinatal adverse events, exposure to psychological stress, paternal age, pre-maternal smoking, low birth weight, medication exposure *in utero*, and autoimmune reaction to group A β-hemolytic streptococcal infection (PANDAS) represent a risk for TS (2, 3).

The most recent heritability studies for TS estimate the overall genetic contribution to be between 58 and 77% (4, 5). Genetic association studies on small and large scales have identified a number of chromosomal regions and gene polymorphisms possibly implicated in TS. The candidates for these studies have traditionally been variations in genes involved in (i) the dopaminergic pathway (treatment with dopamine antagonists effectively reduces the manifestation of tics), (ii) the serotonergic system, (iii) the glutamatergic neurotransmitter system (supporting the theory of glutamate and its involvement in Tourette Syndrome), (iv) the histaminergic system, (v) the gamma-amino butyric acid (GABA) and (vi) the endocannabinoid systems (6).

The most widely studied and validated genes are the dopamine D2 receptor (DRD2), the inner mitochondrial membrane peptidase subunit 2 (IMMP2L) and the SLIT and NTRK Like Family Member 1 (SLITRK1) genes (7). The dopamine D2 and D4 receptors support the dopaminergic theory and are also among the most studied genes in psychiatric disorders in general. The IMMP2L gene encodes a protein involved in processing the signal peptide sequences used to direct mitochondrial proteins to the mitochondria. It has been linked to TS through copy number variation (CNV) studies, which also led to the identification of additional TS associated genes (3), amongst them the arylacetamide deacetylase (AADAC), whose association with TS was further confirmed by a meta-analysis with a large sample size (8).

SLITRK1 is another thoroughly investigated candidate gene in the genetic background of TS after it was first reported by Abelson and colleagues (9). They discovered a frameshift-causing single nucleotide deletion that results in a truncated protein, as well as a further mutation in the 3′ untranslated region (3'UTR) at the putative microRNA (miRNA) binding site for mir-189. It was later linked to TS by other studies, validating the involvement of SLITRK1 in the pathology of the disease (10–13). In a recent study, another 3'UTR targeting miRNA known as mir-429, which is involved midbrain and hindbrain differentiation and synaptic transmission, was found to be under-expressed in the blood serum of TS patients (14).

MiRNAs are small non-coding RNA molecules, which may control gene expression both at transcriptional and post-transcriptional levels via mRNA degradation and/or translational repression. The primary determinant for miRNA binding is a nearly fully congruent, consecutive base-pairing between the target mRNA and the miRNA at position 2–7 in the 5′ end of the mature miRNA. The complementary sequence in the target mRNA is generally located in the 3′UTR (15). Many miRNAs are highly expressed in the brain, where they modulate different biological processes, such as cell survival, neurite projection and synaptic plasticity (16) with a major role in psychiatric disorders (17). In case there is a single nucleotide polymorphism (SNP) within the target binding site of a miRNA, it could result in three possible alterations of miRNA-mediated regulation: (a) it can partially or completely disrupt the miRNA binding site, thus resulting in higher expression of the target gene, or (b) it can enhance the binding affinity of a miRNA to the 3'UTR region through improvement of the original recognition site, or (c) it may create a novel binding site for another miRNA (3, 18). Therefore, such SNPs might alter the binding site of miRNAs, thus affecting TS candidate gene expression by disrupting the miRNA-mediated regulation. Based on these assumptions about the 3'UTR polymorphisms, our study is focused on the investigation of the 3'UTR area of TS candidate genes.

## Materials and Methods

### *In silico* Work

We obtained a list of candidate genes implicated in the etiology of TS by performing a literature search using the terms “Tourette Syndrome” or “TS” combined with “gene” or “genetics” on PubMed and additionally using HugeNavigator tool (https://phgkb.cdc.gov/PHGKB/hNHome.action). In order to investigate the possible involvement of miRNA-mediated regulation in TS, we focused our attention on single nucleotide genetic variations in the 3'UTR regulatory sequence of TS candidate genes using the PolymiRTS database (http://compbio.uthsc.edu/miRSNP/). Specifically, we screened for SNPs predicted to alter the seed sequence of miRNAs in the 3'UTR of our candidate genes. Based on the *in silico* findings, we created a list of presumptive SNPs in the 3'UTR of TS susceptibility genes. The final list of 32 SNPs in 18 genes was then created based on the minor allele frequencies (MAFs) and on the availability of the TaqMan® assays from Applied Biosystems™ (Thermo Fisher Scientific, Waltham, MA, USA). Primarly, variants with MAF between 5 and 50% were selected (HapMap/1000 Genomes data from www.ncbi.nlm.nih.gov). Thirtytwo SNPs were selected for our customized TaqMan® OpenArray® Genotyping plates (Thermo Fisher Scientific) (Table 1).

**Table 1:** SNPs selected for OpenArray® genotyping.

**Gene symbol**	**Gene name**	**SNP**	**Minor allele frequency**	**SNP found in PolymiRTS**
AADAC	Arylacetamide deacetylase	rs1042201	0.49 (A)	
ACP1	Acid phosphatase 1, soluble	rs6855	0.20 (G)	Yes
CNR1	Cannabinoid receptor 1	rs4707436	0.24 (A)	Yes
		rs806368	0.28 (C)	Yes
CNTNAP2	Contactin associated protein-like 2	rs1062072	0.46 (A)	Yes
		rs2530310	0.42 (T)	Yes
		rs2530311	0.47 (A)	Yes
		rs987456	0.30 (C)	Yes
COMT	Catechol-O-methyltransferase	rs165599	0.47 (G)	Yes
		rs165728	0.16 (C)	
DRD2	Dopamine receptor D2	rs6276	0.48 (T)	Yes
		rs6278	0.20 (A)	Yes
GDNF	Glial cell derived neurotrophic factor	rs2973051	0.34 (C)	Yes
		rs3749692	0.46 (A)	Yes
		rs62360370	0.04 (A)	Yes
HTR2A	5-hydroxytryptamine receptor 2A	rs3125	0.14 (G)	Yes
ILR1N	Interleukin 1 receptor antagonist	rs4252041	0.02 (T)	Yes
IMMP2L	Inner mitochondrial membrane peptidase subunit 2	rs1044729	0.34 (C)	
		rs17158195	0.13 (T)	
		rs7795011	0.46 (G)	
LHX6	LIM homeobox 6	rs3750486	0.08 (A)	Yes
		rs74370188	0.01 (A)	Yes
MAOA	Monoamine oxidase A	rs3027407	0.40 (A)	Yes
MEIS1	Meis homeobox 1	rs72824830	0.02 (G)	Yes
MIR302A	MicroRNA 302a	rs114318553	0.004 (G)	
NTN4	Netrin 4	rs1052651	0.38 (A)	Yes
		rs8699	0.43 (G)	Yes
SLC6A3	Solute carrier family 6 member 3	rs11564774	0.23 (G)	
		rs7732456	0.07 (C)	
SLITRK1	SLIT and NTRK like family member 1	rs3737193	0.03 (G)	Yes
		rs41557622	0.02 (T)	Yes
TNF	Tumor necrosis factor	rs3093665	0.02 (C)	Yes

### Samples

DNA samples are part of the TSGeneSEE (Tourette Syndrome Genetics—Southern and Eastern Europe Initiative) consortium collection, which collected samples from patients with a primary diagnosis of Tourette Syndrome from Hungary, Italy, Poland, Greece and Albania. Our dataset is composed of 290 samples (162 TS patients and 128 healthy matched controls) and 444 parent-child trios from 148 TS families.

Specifically, the samples used in the Transmission Disequilibrium Test (TDT) consisted of “complete trios,” where we have records of the complete family (child, mother, and father) and of “incomplete trios,” where one of the parents was missing from the sample. The combined dataset consisting of complete and incomplete trios will be referred to as “families” in the Tables presented in the Results section. Diagnosis of TS was ascertained according to DSM-IV-TR criteria in participants from Italy, Hungary, Albania and Greece, and according to DSM-IV in Polish individuals. Collection of TSGeneSEE samples was coordinated and approved by the Democritus University of Thrace Research Ethics Committee and written informed consent was obtained from all participating individuals or their parents. The average age of onset of the children (154 male, 152 female) involved in the study was 5.7 ± 2.19 years.

### Genotyping

Genotyping of samples was performed by using TaqMan® OpenArray® Genotyping System (19). The processing of raw data was performed by the TaqMan® Genotyping Software (Applied Biosystems, Thermo Fisher Scientific). As a quality control step, 10% of samples were re-genotyped to avoid any batch effects. In addition, samples that failed to pass the genotyping success rate of 90% were removed from the analysis. Thus, the final analysis was performed on 141 families (Table 2) and 266 samples (141 cases, 125 controls) (Supplementary Table 1). Out of the 32 SNPs considered for assessment, 30 SNPs were successfully genotyped. The raw genotyping data are provided in Supplementary Table 2.

**Table 2:** TDT dataset of families Distribution of the dataset across different populations after quality control.

**Population**	**Trios**	**Incomplete trios**	**Full dataset (Families)**
Albania	1	2	3
Greece	9	10	19
Hungary	19	12	31
Italy	40	16	56
Poland	23	9	32
Total	92	49	141

### Statistical Analysis

Standard case-control association analysis was conducted in PLINK (20) using χ2 test comparing SNP frequencies of cases and controls. As population stratification is one of the major concerns in genome-wide association studies due to cryptic differences in allele frequencies between different populations, we performed basic χ^2^ association with respect to TS, separately for the different populations (Supplementary Table 1).

METAL is a tool widely used for meta-analysis genome wide association study. It is especially appropriate when data from the individual studies shows differences in population stratification, phenotype distribution, gender, or constraints in sharing of individual level data imposed. Meta-analysis results in little or no loss of efficiency compared to a classic genetic association analysis where the dataset does not account for variations between individuals (21). Since our dataset is composed of Southern Eastern European samples, we decided to use METAL in order to correct for the presence of population stratification. As population stratification is one of the major concerns in genome-wide association studies due to cryptic differences in allele frequencies between different populations, we first performed basic χ^2^ association with respect to TS, separately for the five different populations. The data was further meta-analyzed combining the four largest population cohorts (Italy, Poland, Hungary and Greece) using the METAL software in a case-weighted fixed-effect on p-values (21). To correct our results for multiple testing, 100,000 permutations were applied with the Monte Carlo Permutation (MCPerm) method (22).

Family-based association was performed using the family-based Transmission Disequilibrium Test option in PLINK software to seek for association with TS. Families were checked for Mendelian errors and samples with error rate above 10% were discarded from further analyses. Empirical significance levels were generated with PLINK using max (T) permutation methods set to 1,000 permutations.

## Results

### Meta-Analysis

Table 3 shows the top results (*p* < 0.1) from our meta-analysis leading to the identification of two significant SNPs that might represent risk for developing TS. The strongest signal was seen at rs3750486 (*p* = 0.021) in the LHX6 gene, while the other significant SNP was rs7795011 (*p* = 0.029) in the IMMP2L gene. Notably, the findings withstood correction for multiple testing. The full results of meta-analysis are provided in Supplementary Table 3.

**Table 3:** Top hits of the meta-analysis.

**Gene**	**SNP**	**Allele1**	**Allele2**	**Frequency of affected allele**	***p***
LHX6	rs3750486	A	G	0.1075	0.021^*^
IMMP2L	rs7795011	T	G	0.5483	0.029^**^
AADAC	rs1042201	A	G	0.4648	0.077
GDNF	rs2973051	T	C	0.6952	0.095

* *MCPerm p-value = 0.001*.

** *MCPerm p-value = 0.009*.

*Bold values indicates the nominally significant p-values*.

### Transmission Disequilibrium Test (TDT)

Similarly, we performed TDT on parent-child trios within each individual population, as well as for the complete TSGeneSEE dataset (141 families). Table 4 shows TDT results of the top 4 SNPs in our meta-analysis analyzed by population on complete trios (*N* = 91). As there was only one complete trio from Albania, this population was left out from Table 4.

**Table 4:** Analyses of the top 4 hits identified by the meta-analysis in the TDT test performed on complete trios.

		**Poland (23 trios)**				**Italy (40 trios)**				**Hungary (19 trios)**				**Greece (9 trios)**			
**GENE**	**SNP**	**Over Ts.^†^**	**T:U^‡^**	**χ2^§^**	***p***	**Over Ts.**	**T:U**	**χ2**	***p***	**Over Ts.**	**T:U**	**χ2**	***p***	**Over Ts.**	**T:U**	**χ2**	***p***
AADAC	rs1042201	G	9:8	0.059	0.808	G	27:13	4.9	**0.027**		5:5	0	1	A	3:0	3	0.083
GDNF	rs2973051	C	10:8	0.222	0.637	C	18:16	0.118	0.732	T	8:7	0.067	0.796	C	6:3	1	0.317
IMMP2L	rs7795011	G	11:7	0.889	0.346	G	25:16	1.976	0.156	G	7:6	0.077	0.782	T	8:0	8	0.005^**^
LHX6	rs3750486	A	5:0	5	**0.025**	G	15:2	9.941	0.002^*^	A	1:0	1	0.317	G	1:0	1	0.317

† *Over Ts., over-transmitted allele*;

‡ *T:U, transmitted-untransmitted ratio*;

§ *χ2, Chi-square value*;

* *permutation p-value = 0.037*;

** *permutation p-value = 0.027*.

*Bold values indicates the nominally significant p-values*.

When analyzing individual populations, rs3750486 in LHX6 showed a slight, but non-significant over-transmission of the A allele in the Polish population (*p* = 0.025, χ^2^ = 5.0) and a significant transmission of the G allele in the Italian population (*p* = 0.002, χ^2^ = 9.941), which remained significant even after 1,000 permutations test (*p* = 0.037). For rs7795011 in IMMP2L, we observed a nominally significant over-transmission of T allele in the Greek population (*p* = 0.005, χ^2^ = 8.0), which withstands 1,000 permutations test (*p* = 0.027). For rs1042201 in AADAC we also found nominally significant over-transmission in the Italian population (*p* = 0.027, χ^2^ = 4.9).

Table 5 shows TDT results of these top 4 SNPs in our meta-analysis of the full TSGeneSEE dataset (all 141 families). The full results of TDT test are provided in Supplementary Table 4.

**Table 5:** TDT test performed on the full TSGeneSEE dataset (141 families).

**Gene**	**SNP**	**Over Ts.^†^**	**T:U^‡^**	**χ2^§^**	***p***
AADAC	rs1042201	A	31:16	4.787	**0.029**
GDNF	rs2973051	C	41:37	0.205	0.650
IMMP2L	rs7795011	G	44:39	0.301	0.583
LHX6	rs3750486	G	7:3	1.6	0.205

† *Over Ts., over-transmitted allele*;

‡ *T:U, transmitted-untransmitted ratio*;

§ *χ2, Chi-square value*.

*Bold values indicates the nominally significant p-values*.

Looking at the top results of the TDT test of the full dataset, only allele A of rs1042201 in AADAC was found to be over-transmitted to TS patients (*p* = 0.029, χ^2^ = 4.787), which is also among the top signals of our TDT analysis done on complete trios. Summing up the results, the AADAC gene is the strongest hit of this TDT study, however the signal did not survive correction for multiple testing.

## Discussion

This is a preliminary study to investigate genetic variation in the 3'UTR of candidate genes in a sample of TS patients, families and control individuals from five Southern and Eastern European populations. In the meta-analysis we found a significant association of rs3750486 (*p* = 0.021, MCPerm *p* = 0.001) located in the 3'UTR region of the LIM homeobox 6 (LHX6) gene with TS. This gene encodes for a member of a large protein family that contains the LIM domain, a unique cysteine-rich zinc-binding domain. This protein is expressed in the developing and adult mouse basal ganglia, in a structure from which striatal interneurons originate (23). LHX6 modulates the expression of fast-spiking, parvalbumin-positive and somatostatin-positive interneurons in the cerebral cortex and striatum (24, 25), where these interneurons play a key role in the regulation of striatal activity (26), a region of the brain believed to be involved in TS pathogenesis (27–29). Specifically, they modulate the activity of spiny projection neurons, which belong to the main cell type of the striatum receiving cortical input and providing information to the substantia nigra and globus pallidus. Moreover, LHX6 may also be involved in controlling the differentiation and development of neural and lymphoid cells (30). LHX6 has been previously associated to TS, however the investigated SNPs are not in linkage disequilibrium (D'=0.293, *r*^2^ = 0.004) with the one in the present study, as we focused only on the 3'UTR region (31). Therefore, we believe that we could have detected one of the biologically functional polymorphisms that can alter the gene expression via a mechanism mediated by miRNAs. Indeed, rs3750486 of LHX6 lies in a putative target sequence of several miRNAs (mir-92a, mir-491, mir-5191), whose binding might be disrupted by the presence of the A allele, whereas the presence of the G allele might create a modified sequence rather suitable for other miRNAs (mir-4447, mir-4472). The available information in the literature on these miRNA variants is rather scarce. Interestingly, mir-92a has been already linked to bipolar disorder (32) and proposed as possible biomarker for schizophrenia (33), while mir-491 has been linked to panic disorders (34) and major depression (35). Moreover, mir-447 has been recently detected in the blood of ADHD patients (36), one of the shared comorbidities of TS.

The second suggestive finding of our meta-analysis is rs7795011 (*p* = 0.029, MCPerm *p* = 0.009) in the IMMP2L gene. IMMP2L encodes for a protein involved in processing the signal peptide sequences used to target mitochondrial proteins into the mitochondria. The sudden increase in free radical production from mitochondria triggers formation of a mitochondrial permeability transition pore, resulting in the release of pro-apoptotic proteins. Therefore, mitochondria may play a central role in the pathophysiology of many neurodegenerative diseases by activating apoptotic pathways and eventually cell death (37). Several studies on copy number variations have shown IMMP2L linkage to TS (38–41). Notably, IMMP2L has been associated with other neuropsychiatric disorders, such as autism, ADHD and speech/language disorders (42–44).

The top hit of our TDT analysis is rs1042201 of the AADAC gene (nominal *p* = 0.029), which also showed a trend for association in the meta-analysis. Arylacetamide deacetylase is an enzyme involved in neutral lipid lipolysis, detoxification and drug metabolism and is mainly expressed in the liver, the small intestine, the adrenal glands and the pancreas (17), however, its function in the human brain is still unclear. It has been recently linked to TS by a CNV analysis in a Danish TS cohort, followed by a meta-analysis of over 1,000 TS patients and 100,000 controls, which confirmed the role of AADAC deletions in TS pathogenesis (8). This study also detected the presence of AADAC protein in several regions of the mouse and human brain (hippocampus, corpus callosum and caudate nucleus) previously implicated in TS (45). Interestingly, rs1042201 lies in a putative target binding site of mir-4263, which may play a role in human embryonic stem cell and neural precursor differentiation (46).

SLITRK1, the most widely studied gene in Tourette Syndrome, did not yield a positive finding in this study. However, a strong connection between a genetic variation in the 3'UTR of the gene and microRNA-mediated regulation has been already found and validated by Abelson and colleagues (9). Our result is consistent with many of the genetic replication studies, which investigated the role of SLITRK1 in relation to TS and failed to provide new or replicated positive findings (47–52).

It should be noted that even though our study yielded interesting findings, which may be relevant to the pathogenesis of TS, the power of the present study is limited by the relatively low sample size. This limitation affects not only this study but many of the genetic analyses on small/medium sample sizes. In addition, the complex genetic background of TS likely originates from both common and rare variants as well as from external factors that severely hinder the possibility of having consistent, replicable findings (3). Moreover, the heterogeneity of dataset represents another limitation of the study. Indeed, although TDT analyses are robust to population stratification (53), it is notable that the results show some differences between populations, which could arise either from stochastic factors and demographic history or pressure from natural selection. It is therefore important to conduct more studies on different populations, especially those that are generally underrepresented in mainstream genetic studies, such as many Eastern European that are featured in the present paper. It is possible that the variety of genetic factors propelling the development of TS might also differ based on the geographic region, as observed in our study. This population difference might help to explain the lack of consistency between different studies.

To overcome this issue, we decided to strengthen our study by focusing solely on the 3'UTR of TS candidate genes given the possibility that a variation in those SNPs might affect the predicted binding of microRNAs thus having a biological repercussion on gene expression via microRNA regulation.

In conclusion, we identified a positive association between rs1042201 in AADAC gene and a significant association between rs3750486 in the LHX6 and rs7795011 in the IMMP2L genes with Tourette Syndrome, thus providing support for their possible biological role in the etiology of the disorder. We speculate that some of those influences may arise from miRNA-mediated modulation of gene expression, however further association studies on larger populations, as well as functional validation follow-up studies are warranted.

## Data Availability Statement

The datasets presented in this study can be found in online repositories. The names of the repository/repositories and accession number(s) can be found in the article/Supplementary Material.

## Ethics Statement

Collection of TSGeneSEE samples was coordinated and approved by the Democritus University of Thrace Research Ethics Committee and written informed consent was obtained from all participating individuals or their parents. GJ: Data check flagged [type] (snp).

## Author Contributions

LP, AV, and CB were involved in the design of the study. PP coordinated the sample collection. ZT, LF, PN, RR, TW, US, MK, EB, AK, CA, VT, and IK contributed to clinical assessment and collection of DNA samples from TS patients and families. LP and AV performed the experiments. LP and SP analyzed the data. LP, SP, and CB wrote the manuscript. All authors contributed to formulation of the final draft of the manuscript and approved the final version.

## Conflict of Interest

The authors declare that the research was conducted in the absence of any commercial or financial relationships that could be construed as a potential conflict of interest.
