# Supplementary material for: Association of Genetic Variation in the 3'UTR of LHX6, IMMP2L, and AADAC With Tourette Syndrome
Source: Front Neurol. 2020 Aug 14;11:803. doi: 10.3389/fneur.2020.00803 (PMC7457023; doi:10.3389/fneur.2020.00803)
Supplement: Supplementary file 1 [file Table_1.DOCX]

| **Supplementary Table 1:**  Sample size distribution of cases and controls across the different populations after quality control. | | |
| --- | --- | --- |
| **Population** | **Cases** | **Controls** |
| Greece | 16 | 29 |
| Hungary | 24 | 29 |
| Italy | 69 | 48 |
| Poland | 32 | 19 |
| Total | 141 | 125 |
