# Supplementary material for: Association of Genetic Variation in the 3'UTR of LHX6, IMMP2L, and AADAC With Tourette Syndrome
Source: Front Neurol. 2020 Aug 14;11:803. doi: 10.3389/fneur.2020.00803 (PMC7457023; doi:10.3389/fneur.2020.00803)
Supplement: Supplementary file 3 [file Table_3.DOCX]

| **Supplementary Table 3: Full results of meta-analysis** | | | | | | |
| --- | --- | --- | --- | --- | --- | --- |
| Gene | SNP | Allele1 | Allele2 | Freq^†^ | p-value^‡^ | MCPerm p-value^§^ |
| LHX6 | rs3750486 | a | g | 0.1075 | **0.02118** | **0.00136** |
| IMMP2L | rs7795011 | t | g | 0.5483 | **0.02903** | **0.00935** |
| AADAC | rs1042201 | a | g | 0.4648 | 0.0767 | 0.22116 |
| GDNF | rs2973051 | t | c | 0.6952 | 0.09461 | 0.557 |
| HTR2A | rs3125 | c | g | 0.8768 | 0.09844 | 0.49278 |
| CNR1 | rs4707436 | a | g | 0.2260 | 0.112 | 0.26288 |
| IL1RN | rs4252041 | t | c | 0.0588 | 0.1648 | 0.8822 |
| COMT | rs165728 | t | c | 0.9307 | 0.1931 | 0.2523 |
| COMT | rs165599 | a | g | 0.7596 | 0.2307 | 0.82317 |
| SLC6A3 | rs11564774 | c | g | 0.7381 | 0.2308 | 0.57694 |
| CNTNAP2 | rs2530310 | t | c | 0.4202 | 0.3448 | 0.62349 |
| IMMP2L | rs1044729 | t | c | 0.6722 | 0.3565 | 0.39905 |
| IMMP2L | rs17158195 | a | t | 0.9424 | 0.3794 | 0.68283 |
| SLITRK1 | rs41557622 | a | t | 0.9571 | 0.3818 | 0.03628 |
| DRD2 | rs6278 | a | c | 0.1346 | 0.3931 | 0.46421 |
| GDNF | rs62360370 | a | g | 0.0856 | 0.4178 | 0.41403 |
| CNR1 | rs806368 | t | c | 0.7585 | 0.4358 | 0.15785 |
| MEIS1 | rs72824830 | a | g | 0.9844 | 0.4876 | 0.7239 |
| LHX6 | rs74370188 | a | g | 0.0347 | 0.5102 | 0.00646 |
| DRD2 | rs6276 | t | c | 0.7089 | 0.5956 | 0.53492 |
| TNF | rs3093665 | a | c | 0.9518 | 0.6009 | NA |
| ACP1 | rs6855 | a | g | 0.8356 | 0.6901 | 0.90212 |
| GDNF | rs3749692 | a | g | 0.4178 | 0.7064 | 0.26411 |
| NTN4 | rs1052651 | a | g | 0.4381 | 0.7715 | 0.04658 |
| NTN4 | rs8699 | a | g | 0.6164 | 0.8051 | 0.01123 |
| CNTNAP2 | rs987456 | a | c | 0.8376 | 0.8366 | 0.03142 |
| SLITRK1 | rs3737193 | a | g | 0.9541 | 0.8801 | 0.7077 |
| SLC6A3 | rs7732456 | a | c | 0.9384 | 0.8893 | 0.61032 |
| CNTNAP2 | rs1062072 | a | g | 0.4725 | 0.933 | 0.02471 |
| CNTNAP2 | rs2530311 | a | g | 0.4851 | 0.9457 | 0.04518 |

†Freq: frequency of the affected allele, ‡p-value: unadjusted p-value, §MCPerm p-value: Monte Carlo Permutation p-value
