# Supplementary material for: Association of Genetic Variation in the 3'UTR of LHX6, IMMP2L, and AADAC With Tourette Syndrome
Source: Front Neurol. 2020 Aug 14;11:803. doi: 10.3389/fneur.2020.00803 (PMC7457023; doi:10.3389/fneur.2020.00803)
Supplement: Supplementary file 4 [file Table_4.DOCX]

| **Supplementary Table 4: Results of the TDT results on the full TSGeneSEE dataset (141 families)** | | | | | | | | | | |
| --- | --- | --- | --- | --- | --- | --- | --- | --- | --- | --- |
| Gene | CHR^†^ | SNP | Allele1 | Allele2 | T^‡^ | U^§^ | OR^¶^ | χ2^#^ | p-value^*^ | Perm p-value^**^ |
| AADAC | 3 | rs1042201 | G | A | 16 | 31 | 0.5161 | 4.787 | 0.02867 | 0.524 |
| MEIS1 | 2 | rs72824830 | G | A | 2 | 7 | 0.2857 | 2.778 | 0.09558 | 0.925 |
| IMMP2L | 7 | rs17158195 | T | A | 8 | 16 | 0.5 | 2.667 | 0.1025 | 0.957 |
| HTR2A | 13 | rs3125 | G | C | 14 | 24 | 0.5833 | 2.632 | 0.1048 | 0.966 |
| TNF | 6 | rs3093665 | C | A | 5 | 11 | 0.4545 | 2.25 | 0.1336 | 0.980 |
| LHX6 | 9 | rs3750486 | A | G | 3 | 7 | 0.4286 | 1.6 | 0.2059 | 0.999 |
| LHX6 | 9 | rs74370188 | A | G | 3 | 7 | 0.4286 | 1.6 | 0.2059 | 0.999 |
| CNTNAP2 | 7 | rs1062072 | C | T | 52 | 40 | 1.3 | 1.565 | 0.2109 | 0.999 |
| CNTNAP2 | 7 | rs2530311 | A | G | 53 | 43 | 1.233 | 1.042 | 0.3074 | 1 |
| CNTNAP2 | 7 | rs2530310 | A | G | 53 | 43 | 1.233 | 1.042 | 0.3074 | 1 |
| CNTNAP2 | 7 | rs987456 | C | A | 29 | 22 | 1.318 | 0.9608 | 0.327 | 1 |
| CNR1 | 6 | rs806368 | A | G | 47 | 38 | 1.237 | 0.9529 | 0.329 | 1 |
| CNR1 | 6 | rs4707436 | C | T | 32 | 40 | 0.8 | 0.8889 | 0.3458 | 1 |
| DRD2 | 11 | rs6278 | A | C | 21 | 16 | 1.312 | 0.6757 | 0.4111 | 1 |
| GDNF | 5 | rs62360370 | A | G | 10 | 14 | 0.7143 | 0.6667 | 0.4142 | 1 |
| IMMP2L | 7 | rs1044729 | C | T | 36 | 30 | 1.2 | 0.5455 | 0.4602 | 1 |
| SLITRK1 | 13 | rs41557622 | T | A | 7 | 10 | 0.7 | 0.5294 | 0.4669 | 1 |
| IL1RN | 2 | rs4252041 | T | C | 7 | 5 | 1.4 | 0.3333 | 0.5637 | 1 |
| COMT | 22 | rs165599 | G | A | 24 | 28 | 0.8571 | 0.3077 | 0.5791 | 1 |
| IMMP2L | 7 | rs7795011 | G | T | 44 | 39 | 1.128 | 0.3012 | 0.5831 | 1 |
| GDNF | 5 | rs3749692 | A | G | 51 | 46 | 1.109 | 0.2577 | 0.6117 | 1 |
| GDNF | 5 | rs2973051 | C | T | 41 | 37 | 1.108 | 0.2051 | 0.6506 | 1 |
| SLC6A3 | 5 | rs11564774 | G | C | 25 | 27 | 0.9259 | 0.07692 | 0.7815 | 1 |
| SLITRK1 | 13 | rs3737193 | G | A | 6 | 7 | 0.8571 | 0.07692 | 0.7815 | 1 |
| NTN4 | 12 | rs8699 | G | A | 42 | 44 | 0.9545 | 0.04651 | 0.8292 | 1 |
| NTN4 | 12 | rs1052651 | A | G | 44 | 46 | 0.9565 | 0.04444 | 0.833 | 1 |
| ACP1 | 2 | rs6855 | A | G | 32 | 31 | 1.032 | 0.01587 | 0.8997 | 1 |
| DRD2 | 11 | rs6276 | C | T | 33 | 34 | 0.9706 | 0.01493 | 0.9028 | 1 |
| SLC6A3 | 5 | rs7732456 | C | A | 11 | 11 | 1 | 0 | 1 | 1 |
| COMT | 22 | rs165728 | C | T | 9 | 9 | 1 | 0 | 1 | 1 |

†CHR: chromosome, ‡T: transmitted, §U:untransmitted, ¶OR: odds ratio, #χ2: TDT chi-square value, *p-value: unadjusted p-value, **Perm p-value: 1,000 permutations p-value
